# HCCD-DS v2: a transparent synthetic benchmark for human–AI decision support under contextual uncertainty

**DOI:** 10.1038/s41598-026-59232-0

**Published:** 2026-06-24

**Authors:** Kadir Kesgin

**Affiliations:** Department of Computer Technologies, Gönen Vocational School, Bandırma Onyedi Eylül University, Balıkesir, Turkey

**Keywords:** Decision support systems, Human–AI interaction, Benchmark dataset, Explainable artificial intelligence, Trust calibration, Reproducible evaluation, Computational biology and bioinformatics, Health care, Mathematics and computing

## Abstract

Decision support pipelines increasingly combine machine learning predictions with human judgment, yet most public benchmarks evaluate model outputs only and do not encode the interaction process that determines final decisions. This limits reproducible analysis of when human intervention improves or degrades system-level performance. We introduce HCCD-DS v2, a transparent and configurable synthetic benchmark dataset that models decision-time interaction between an AI recommender and simulated users with continuous expertise levels under contextual uncertainty. Each decision instance links input context, system confidence, explainability metadata, human acceptance/override behavior, override rationale, and realized outcome. The dataset simulates distinct profiles for healthcare, cybersecurity, and Internet of Things (IoT) scenarios to support cross-domain evaluation. Empirical analyses and benchmark experiments show that human intervention is condition-dependent: expert overrides are more likely to improve outcomes, while novice overrides more often reduce success. By exposing this interaction in a unified and reusable resource under explicit behavioral assumptions (sequential trust updates, individual risk tolerance, and explanation sensitivity), HCCD-DS v2 provides a controlled environment for benchmarking trust calibration, explanation-aware interaction policies, and algorithms that learn to defer. We discuss the limits of predictive recoverability in simulated human behavior and frame the benchmark’s F1-score transitions as a measure of policy complexity.

## Introduction

Decision Support Systems (DSS) are increasingly implemented as hybrid pipelines in which machine learning models generate recommendations and humans decide whether to follow or override them. In this setting, system-level performance depends on interaction dynamics rather than model accuracy alone. However, most publicly used benchmarks remain prediction-centric and provide limited support for evaluating human–AI decision behavior^1–3^.

This benchmark gap is now a technical bottleneck. In operational environments such as healthcare, cybersecurity, and IoT management, practitioners must decide under uncertainty, time pressure, and incomplete information. These conditions directly affect trust, reliance, and override frequency, but they are rarely encoded in standard datasets^4,5^. As a result, many methods can be compared on predictive metrics, while few can be compared on interaction-aware decision quality.

Prior work in human-in-the-loop and interactive machine learning has shown that human intervention is heterogeneous: domain experts may correct model failures, whereas low-expertise users may introduce additional errors through unnecessary overrides^6–8^. Yet reproducible, decision-level datasets that jointly represent context, recommendation confidence, explanation signals, user action, and final outcome remain scarce^9–11^.

To address this need, we present HCCD-DS v2, a Human-Centric Context-Aware Decision Support Dataset designed as a reusable benchmark for interaction-aware evaluation. HCCD-DS v2 links contextual uncertainty, system confidence, continuous user expertise, dynamic trust states, individual risk tolerance, decision override behavior, override rationale, explainability metadata, and outcome-level effects in a unified schema. The dataset spans healthcare, cybersecurity, and IoT scenarios and is accompanied by reproducible analysis and benchmark protocols, enabling direct comparison of methods for human-aware decision support^12^.

To clarify the positioning of HCCD-DS v2 within the broader DSS and human–AI interaction literature, Table 1 provides a conceptual comparison between commonly used dataset categories and the proposed dataset. The comparison highlights how existing benchmarks typically focus on isolated aspects of decision-making, whereas HCCD-DS v2 integrates contextual uncertainty, human behavior, system confidence, and explainability within a unified framework.

**Table 1:** Conceptual comparison between HCCD-DS v2 and existing dataset categories.

Characteristic	Traditional DSS	ML benchmarks	HITL logs	HCCD-DS v2
Contextual uncertainty modeled	No	Partial	Partial	Yes
System confidence recorded	No	Yes	Yes	Yes
Human decision captured	No	No	Yes	Yes
Expertise differentiation	No	No	Partial	Yes
Continuous expertise score	No	No	No	Yes
Sequential trust dynamics	No	No	No	Yes
Override reason annotated	No	No	No	Yes
Outcome-level success modeled	Partial	Yes	Partial	Yes
Explainability metadata	No	Partial	No	Yes
Multi-domain applicability	Partial	Yes	No	Yes

The primary contribution of HCCD-DS v2 is the explicit integration of recommendation, interaction, and outcome signals within a single benchmark resource. This design supports reproducible evaluation of methods that model when intervention should occur, which interventions are likely to be beneficial, and how confidence and explanation cues relate to downstream decision quality.

## Related work

Decision Support Systems (DSS) have been studied extensively for several decades, with early work focusing on structured decision problems, rule-based inference, and optimization-oriented frameworks. Classical DSS architectures aimed to support managerial and organizational decision-making by integrating data management, analytical models, and knowledge-based components^1,3^. While these systems emphasized transparency and logical consistency, they were often limited in their ability to cope with uncertainty, dynamic environments, and incomplete information^13^.

The incorporation of machine learning and data analytics has significantly reshaped modern DSS research. Contemporary AI-driven DSS increasingly rely on predictive models, pattern recognition, and automated decision policies to handle large-scale and high-dimensional data^2,14^. Benchmark datasets in this line of research typically prioritize classification accuracy, forecasting performance, or optimization efficiency^15^. While these datasets are valuable for evaluating algorithmic improvements, they implicitly assume that system outputs are either directly actionable or uniformly trusted by human operators, an assumption that rarely holds in practice^16^.

In real-world applications, DSS recommendations are rarely executed in isolation. Human decision-makers actively interpret system outputs, weigh contextual factors, and exercise judgment based on experience and domain knowledge^8,9^. This observation has motivated growing interest in *human-in-the-loop* and *interactive machine learning* paradigms, which argue that effective AI systems should complement rather than replace human expertise^6,7,17^.

Despite this conceptual shift, empirical research on human–AI collaboration remains constrained by data availability. Many studies rely on controlled user experiments, limited observational datasets, or proprietary system logs that are not publicly accessible^5,18^. While such approaches provide valuable insights, they limit reproducibility and hinder systematic comparison across domains and decision contexts. Moreover, existing interaction logs often record user actions without explicitly modeling contextual uncertainty, expertise levels, or structured override rationales, making it difficult to analyze why and under what conditions humans intervene^19^.

Another closely related research stream concerns trust and reliance in AI-assisted decision-making. Prior studies have shown that user trust is influenced not only by system accuracy but also by confidence estimates, transparency, prior experience, and perceived accountability^16,20^. Over-reliance on automated systems can lead to automation bias, whereas excessive distrust may result in unnecessary overrides and degraded performance^21^. Although these phenomena are well documented conceptually, datasets that explicitly capture trust-related behavior at the decision-instance level remain scarce^5^.

Explainable Artificial Intelligence (XAI) has emerged as a complementary research direction aimed at improving transparency and accountability in DSS. Model-agnostic explanation techniques such as SHAP and counterfactual explanations provide insights into feature importance and model reasoning^22,23^. While XAI methods are increasingly applied in empirical studies, most datasets treat explainability as a post-hoc analytical tool rather than an integral component of the decision record^10,11^. Consequently, the relationship between explanations, human trust, and decision outcomes remains difficult to study systematically^24^.

More recently, several works have emphasized the importance of modeling contextual uncertainty and behavioral factors in the evaluation of DSS and human–AI systems^4,12^. Nevertheless, available datasets remain fragmented, domain-specific, or focused on isolated aspects of the decision process, such as system outputs or user feedback. To date, no publicly available dataset provides a unified representation of contextual uncertainty, system confidence, human expertise, decision overrides, outcome-level effects, and explainability metadata within a single, reproducible framework.

Against this backdrop, HCCD-DS v2 is positioned as a response to a clear gap in the literature. Rather than emphasizing predictive performance alone, the dataset is explicitly designed to capture decision-making behavior under behaviorally plausible conditions, where human expertise, contextual uncertainty, and system confidence jointly shape outcomes. By integrating these dimensions across multiple domains, HCCD-DS v2 enables systematic investigation of human–AI collaboration, trust calibration, and explainable decision support in a manner not supported by existing benchmarks.

**Table 2:** Conceptual positioning of HCCD-DS v2 with respect to major research directions.

Research focus	Context	Human factors	Explainability	Outcomes
Traditional DSS studies	Limited	Implicit	Limited	Partial
ML-Based DSS benchmarks	Partial	Absent	Partial	Strong
Human-in-the-Loop Studies	Limited	Strong	Limited	Partial
Trust & XAI-Oriented Studies	Limited	Partial	Strong	Limited
HCCD-DS v2 (Proposed)	Strong	Strong	Yes	Strong

Table 2 provides a conceptual overview of how major research directions in the DSS literature differ in terms of their analytical focus and behavioral coverage. Traditional DSS studies primarily emphasize structured decision logic and outcome evaluation, often treating human judgment as an implicit or external component^1^. In contrast, machine learning–based DSS benchmarks offer strong support for outcome-level performance analysis but typically abstract away human decision behavior and contextual uncertainty^2^.

Human-in-the-loop research explicitly addresses user interaction and decision intervention; however, these studies are frequently limited to controlled experiments or domain-specific applications, with restricted contextual modeling and limited outcome generalization^6,7^. Similarly, trust- and explainability-oriented studies focus on transparency and user reliance, yet often analyze explanations as post-hoc artifacts rather than as integrated elements of the decision process^16,22^.

In comparison, HCCD-DS v2 is designed to unify these previously fragmented perspectives within a single dataset. By jointly modeling contextual uncertainty, human decision behavior, explainability metadata, and outcome-level effects across multiple domains, the dataset enables systematic investigation of decision-making behavior under behaviorally plausible human–AI collaboration settings. This positioning highlights the role of HCCD-DS v2 not as a replacement for existing benchmarks, but as a complementary resource that supports behavioral, trust-aware, and context-sensitive DSS research Table 3.

**Table 3:** Positioning of HCCD-DS v2 against common benchmark categories.

Benchmark category	Domain	Human action	Conf.	Explain.	Outcome	Main limitation or contribution
Representative DSS benchmarks	Single task or domain	No	Partial	Partial	Yes	Strong predictive evaluation, but limited interaction-aware variables.
Representative HITL logs	Platform-specific	Yes	Partial	No/Partial	Partial	Capture user actions, but usually miss a unified uncertainty, confidence, explanation, and outcome schema.
HCCD-DS v2 (this work)	Healthcare, cybersecurity, IoT	Yes	Yes	Yes	Yes	Reusable benchmark linking context, confidence, human intervention, override rationale, explanations, and outcomes.

## Materials and methods

The HCCD-DS v2 dataset was constructed with the explicit objective of modeling *decision-making behavior* rather than isolated predictive performance. The primary design goal was to capture how contextual uncertainty, system-generated recommendations, and human expertise jointly influence final decisions and outcomes in behaviorally plausible decision support system (DSS) settings. To achieve this, a hybrid simulation framework was developed that represents the decision lifecycle as a sequence of interacting components reflecting contemporary human–AI collaborative environments.

Each record in the dataset corresponds to a single decision instance composed of four sequential stages: contextual scenario generation, system recommendation modeling, human-in-the-loop decision simulation, and outcome realization with explainability metadata. This pipeline-based structure ensures reproducibility while allowing behavioral variability to emerge naturally from interactions between system confidence, contextual conditions, and human expertise.

**Fig. 1:**
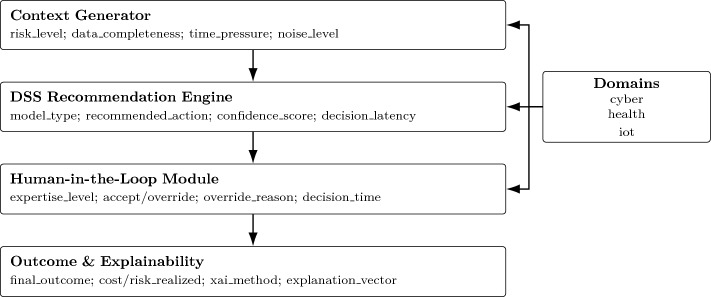
Overview of the HCCD-DS v2 dataset construction pipeline. Each decision instance is generated through contextual scenario synthesis, DSS recommendation modeling, human-in-the-loop behavior simulation, and outcome realization with decision-level explainability metadata.

An overview of the dataset construction process is illustrated in Figure 1. The figure summarizes how contextual factors are transformed into system recommendations, how these recommendations are evaluated by human decision-makers, and how final outcomes and explanations are generated.

To ensure methodological transparency and reproducibility, we summarize the key parameters and literature-informed behavioral rationales of the HCCD-DS v2 framework in Table 4.

**Table 4:** Key generation parameters and literature-informed behavioral rationales for HCCD-DS v2.

Parameter	Value/Range	Behavioral interpretation	Literature-informed rationale	Sensitivity range
$\eta _{+}$	0.05	Trust building step size	Represents incremental trust growth after successful AI recommendation^20^.	[0.01, 0.12]
$\eta _{-}$	0.15	Trust decay step size	Captures algorithm aversion: failures degrade trust $\sim 3\times$ faster than successes build it^16^.	[0.05, 0.35]
$E_j$	$\text {Beta}(2,2) \in [0,1]$	Continuous expertise score	Models continuous skill variability across a user pool, improving ecological validity^9^.	[0, 1]
$RT_j$	$\mathcal {N}(0.5, 0.15)$	Individual risk tolerance bias	Controls sensitivity to contextual risk: low tolerance triggers cautious overrides under high risk.	[0.10, 0.90]
*ε*	0.02	Random baseline override rate	Accounts for routine check-offs, policy-driven overrides, or accidental inputs^21^.	[0.01, 0.08]
$N_{users}$	50	Number of simulated practitioners	Simulates a localized department team interacting sequentially with the DSS.	[10, 200]
$N_{instances}$	15,000	Total benchmark instances	Large-scale sample sizes for stable model training and validation.	[5,000, 50,000]

The core sequence of the decision generation mechanism in HCCD-DS v2 is formalized in Algorithm 1. The procedure details how contextual features are transformed into system recommendations, how explanation quality calibrates trust, and how trust adapts dynamically based on outcomes.

**Algorithm 1:**
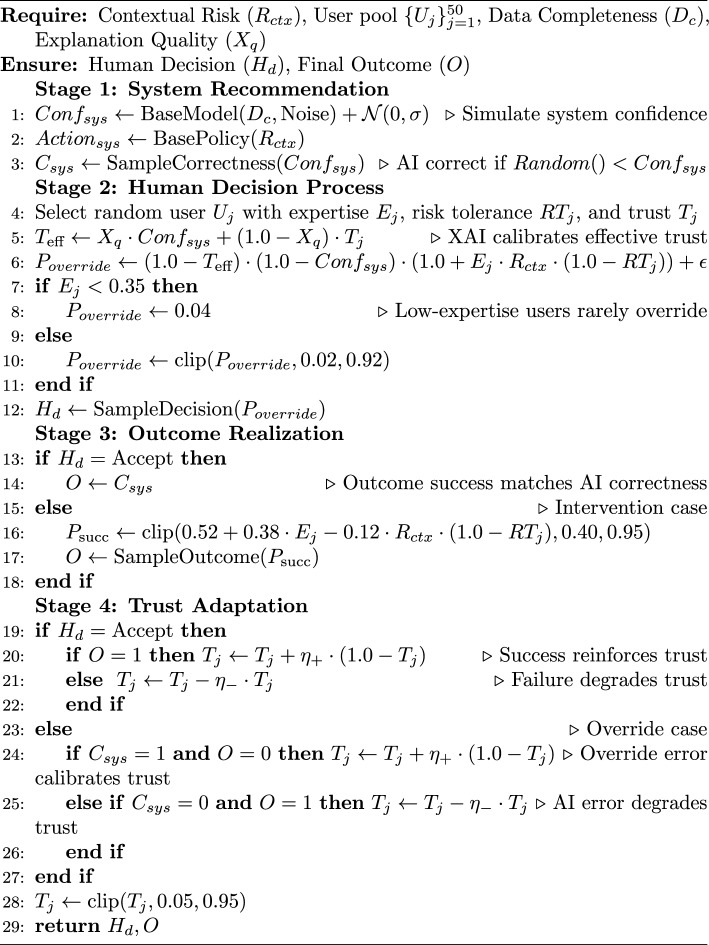
HCCD-DS v2 decision generation logic

The dynamic updates reflect algorithm aversion, ensuring that model failures degrade trust more aggressively than successful outcomes build it. All parameters are exposed and configurable in the benchmark pipeline to support controlled sensitivity sweeps.

### Domain-specific contextual scenario generation

The first stage of the framework generates contextual scenarios intended to reflect heterogeneous and uncertain operating conditions commonly encountered in real-world DSS deployments. Scenarios are generated for three distinct application domains, each characterized by custom action spaces and distinct parameter distributions (as detailed in Table 5):

- *Healthcare*: Characterized by high average risk (modeled as a continuous variable $R_{ctx} \sim \text {Beta}(4,2) \in [0,1]$), low time pressure, high data completeness, and domain-specific actions (*prescribe*, *escalate*, *monitor*).
- *Cybersecurity*: Characterized by moderate average risk ($R_{ctx} \sim \text {Beta}(2.5,3) \in [0,1]$), high time pressure, low data completeness (high noise), and domain-specific actions (*block_ip*, *quarantine*, *allow*).
- *IoT*: Characterized by low average risk ($R_{ctx} \sim \text {Beta}(1.5,5) \in [0,1]$), extremely high time pressure, moderate data completeness, and domain-specific actions (*reboot*, *reconfigure*, *alert*).

Environmental noise levels (low, medium, high) and time pressure (low, medium, high) are determined dynamically based on data completeness and scenario requirements.

### System recommendation and confidence modeling

In the second stage, contextual scenarios are processed by a simulated DSS recommendation engine. The engine generates a recommended action and an associated confidence score $Conf_{sys} \in [0.40, 0.99]$. The base confidence of the system is modeled as 0.82, which is discounted by high noise (-0.18) or low data completeness (-0.12), and slightly reinforced by low noise (+0.04). System confidence is subjected to Gaussian noise $\mathcal {N}(0, 0.08)$ to represent system uncertainty:

$$\begin{aligned} Conf_{sys} = \text {clip}(\text {BaseConf} + \mathcal {N}(0, 0.08), 0.05, 0.99) \end{aligned}$$

The recommendation correctness is sampled probabilistically based on the confidence score. Decision latency is modeled as a stochastic variable following a log-normal distribution to reflect computational variations across domains (e.g., healthcare latency averages 800 ms, while IoT latency is under 20 ms).

### Continuous user profiles and sequential trust adaptation

Unlike static models, HCCD-DS v2 simulates a user pool of ${\mathrm{N}}_{{{\mathrm{users}}}} \; = \;50$ distinct practitioners who sequentially interact with the DSS. Each user *j* is characterized by:

- A continuous expertise score $E_j \in [0, 1]$ sampled from a Beta distribution $\text {Beta}(2,2)$. This continuous score serves as a proxy for the user’s domain skill and directly determines the success of manual interventions.
- An individual risk tolerance bias $RT_j \sim \mathcal {N}(0.5, 0.15)$, which modulates the user’s cautiousness. Users with lower risk tolerance are more likely to override recommendations in high-risk scenarios.
- A dynamically updated sequential trust state $T_{j,t} \in [0.05, 0.95]$. At step *t*, the trust $T_{j,t}$ is updated recursively:
  $$\begin{aligned} T_{j,t} = \text {clip}\left( T_{j,t-1} + \eta _{+}\mathbb {I}(S_t)(1.0 - T_{j,t-1}) - \eta _{-}\mathbb {I}(F_t)T_{j,t-1}, 0.05, 0.95\right) \end{aligned}$$
  where the update coefficients capture algorithm aversion ($\eta _+ = 0.05$ trust gain, $\eta _- = 0.15$ trust decay), $\mathbb {I}(S_t)$ indicates trust reinforcement after successful outcomes (or correct calibration overrides) at step *t-1*, and $\mathbb {I}(F_t)$ indicates trust degradation following model errors (or incorrect user overrides) at step *t-1*.

### Explanation-sensitive human decision simulation

Human decision behavior represents the core behavioral component of HCCD-DS v2. Each decision instance is associated with a simulated human decision-maker from the user pool. The decision to accept or override the AI is generated using a probabilistic rule-based mechanism conditioned on system confidence, contextual risk, explanation quality, user expertise, risk tolerance, and trust:

1. *Explanation Calibration*: High-quality explanations $X_{\mathrm{qual}} \in [0, 1]$ that align with the context calibrate user reliance. The user’s effective trust $T_{\text {eff}}$ is modeled as:
  $$T_{{{\mathrm{eff}}}} = X_{{{\mathrm{qual}}}} \cdot {\mathrm{Conf}}_{{{\mathrm{sys}}}} + (1.0 - X_{{{\mathrm{qual}}}} ) \cdot T_{{j,t}} {\text{ }}$$
2. *Decision Gate*: The probability of overriding the AI recommendation is calculated as:
  $$P_{{{\mathrm{override}}}} = (1.0 - T_{{{\mathrm{eff}}}} ) \cdot (1.0 - {\mathrm{Conf}}_{{{\mathrm{sys}}}} ) \cdot (1.0 + E_{j} \cdot R_{{ctx}} \cdot (1.0 - RT_{j} )) + \varepsilon$$
  where *\varepsilon = 0.02* is a random baseline override rate. Low-expertise users ($E_j < 0.35$) are capped at a low override probability ($P_{\mathrm{override}} = 0.04$) to represent passive compliance.

**Fig. 2:**
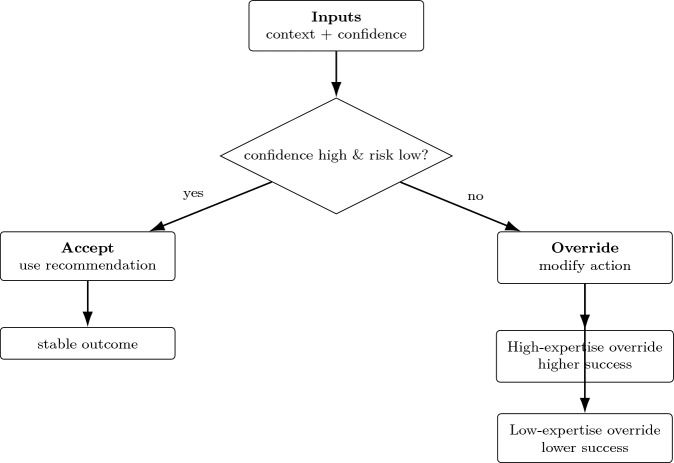
Simplified conceptual illustration of human decision logic. While HCCD-DS v2 uses continuous expertise scores and sequential trust adaptation, this diagram represents the stylized decision gate and resulting outcomes for novice and expert groups.

Figure 2 illustrates this decision gate as a simplified conceptual illustration of the human decision process under continuous expertise and dynamic trust constraints.

### Outcome realization and dataset composition

Final decision outcomes are generated probabilistically. If the user accepts the AI recommendation ($H_d = \text {Accept}$), the outcome success matches the system recommendation correctness ($O = C_{sys}$). If the user overrides the recommendation, the outcome success probability $P_{\text {succ}}$ depends on the user’s continuous expertise score, discounted by scenario risk and individual risk tolerance:

$$\begin{aligned} P_{\text {succ}} = \text {clip}(0.52 + 0.38 \cdot E_j - 0.12 \cdot R_{ctx} \cdot (1.0 - RT_j), 0.40, 0.95) \end{aligned}$$

The final dataset contains 15,000 decision instances split into a 12,000 training split and a 3,000 test split using stratified sampling across healthcare, cybersecurity, and IoT domains.

## Results

This section presents the descriptive analysis and benchmark evaluations of the HCCD-DS v2 dataset. The reported statistics are computed using the synthetic splits (12,000 training and 3,000 test records).

### Descriptive statistics and domain profiles

*Dataset overview and domain balance*. HCCD-DS v2 consists of a total of 15,000 decision instances. The dataset is split into a 12,000 training split and a 3,000 test split. Particular care was taken to avoid dominance by any single application domain. As a result, cybersecurity, healthcare, and IoT scenarios are represented in a balanced manner, with 5,143 cybersecurity (34.29%), 5,012 healthcare (33.41%), and 4,845 IoT (32.30%) instances.

*Domain-specific profiles*. Unlike nominal domain assignments, HCCD-DS v2 implements distinct characteristics for each domain. Table 5 summarizes these characteristics, showing how risk, completeness, and system confidence vary, resulting in distinct override and success behaviors.

**Table 5:** Domain-specific profiles and decision characteristics (full split).

Domain	Mean risk	Mean comp. (%)	Mean conf. (%)	Mean latency	Override rate (%)	Success rate (%)
Healthcare	0.670	84.6	81.3	800 ms	6.54	83.20
Cybersecurity	0.449	70.0	74.3	250 ms	12.41	68.81
IoT	0.228	77.6	77.8	20 ms	8.61	74.96

Time pressure distributions also follow clear domain realities, as illustrated in Table 6. Healthcare decisions are primarily deliberate (low pressure), whereas IoT tasks are heavily constrained (high time pressure).

**Table 6:** Time pressure distribution (%) across domains.

Domain	Low pressure (%)	Medium pressure (%)	High pressure (%)
Healthcare	70.93	18.93	10.14
Cybersecurity	10.25	29.79	59.97
IoT	4.50	15.17	80.33

*Human decision behavior and expertise effects*. A core design objective of HCCD-DS v2 is to encode expertise-driven differences. The dataset captures a clear gradient in override tendencies and rationales. Novice users override system recommendations infrequently (4.28%), with overrides primarily driven by policy (60%) or trust (35%). Intermediate users exhibit a moderate override rate (11.63%), with reasons split between trust and experience. Expert users override system recommendations in 12.03% of cases, with experience being the dominant rationale (70%). The aggregate distribution of override reasons is shown in the manuscript analyses: experience (41.58%), trust (36.95%), and policy (21.48%).

*Outcome-level performance and conditional value of expertise*. The overall success rate of decisions is 75.61%. When system recommendations are accepted by human decision-makers, the success rate is 76.25%. However, the effect of overrides is strongly conditioned on expertise, as documented in Table 7. Expert overrides lead to improved outcomes (success rate of 81.44%), correcting system errors. In contrast, novice overrides substantially reduce success to 49.77%, illustrating the “novice penalty” when overrides are performed without sufficient domain capability.

**Table 7:** Outcome-level success rates by human decision and expertise level.

Expertise level	Accept recommendation (%)	Override recommendation (%)
Novice	74.91	49.77
Intermediate	77.36	67.42
Expert	76.43	81.44
Overall	76.25	69.27

*Correlation analysis* Linear correlation analysis was conducted to examine relationships among selected variables. Expertise level exhibits a weak positive correlation with final success (0.0294), indicating plausible noise and avoiding deterministic assumptions. The correlation between system confidence and contextual risk is 0.1547, showing that system confidence is loosely coupled with environmental risk. User trust and system confidence exhibit a correlation of 0.1478, indicating calibrated trust adaptation.

*XAI trust calibration impact* We evaluate the impact of explanation quality ($X_q$) on human decision-making and trust calibration in Table 8. High-quality explanations are associated with slightly lower override rates and higher acceptance rates, but the observed success differences are small and should be interpreted as calibration effects rather than direct performance gains. The slightly lower success rate in the high-XAI bin reflects domain and risk-composition effects rather than a direct negative explanation effect.

**Table 8:** Impact of explanation quality ($X_q$) on decision and trust metrics.

XAI quality bin	Override rate (%)	Acceptance rate (%)	Success rate (%)	Mean user trust
Low ([0.0, 0.4))	9.719	90.281	75.889	0.501
Medium ([0.4, 0.7))	9.180	90.820	75.999	0.499
High ([0.7, 1.0])	8.436	91.564	74.267	0.496

### Validation and benchmarking

This section presents validation experiments designed to assess internal validity, policy recoverability, and analytical utility of HCCD-DS v2.

**Fig. 3:**
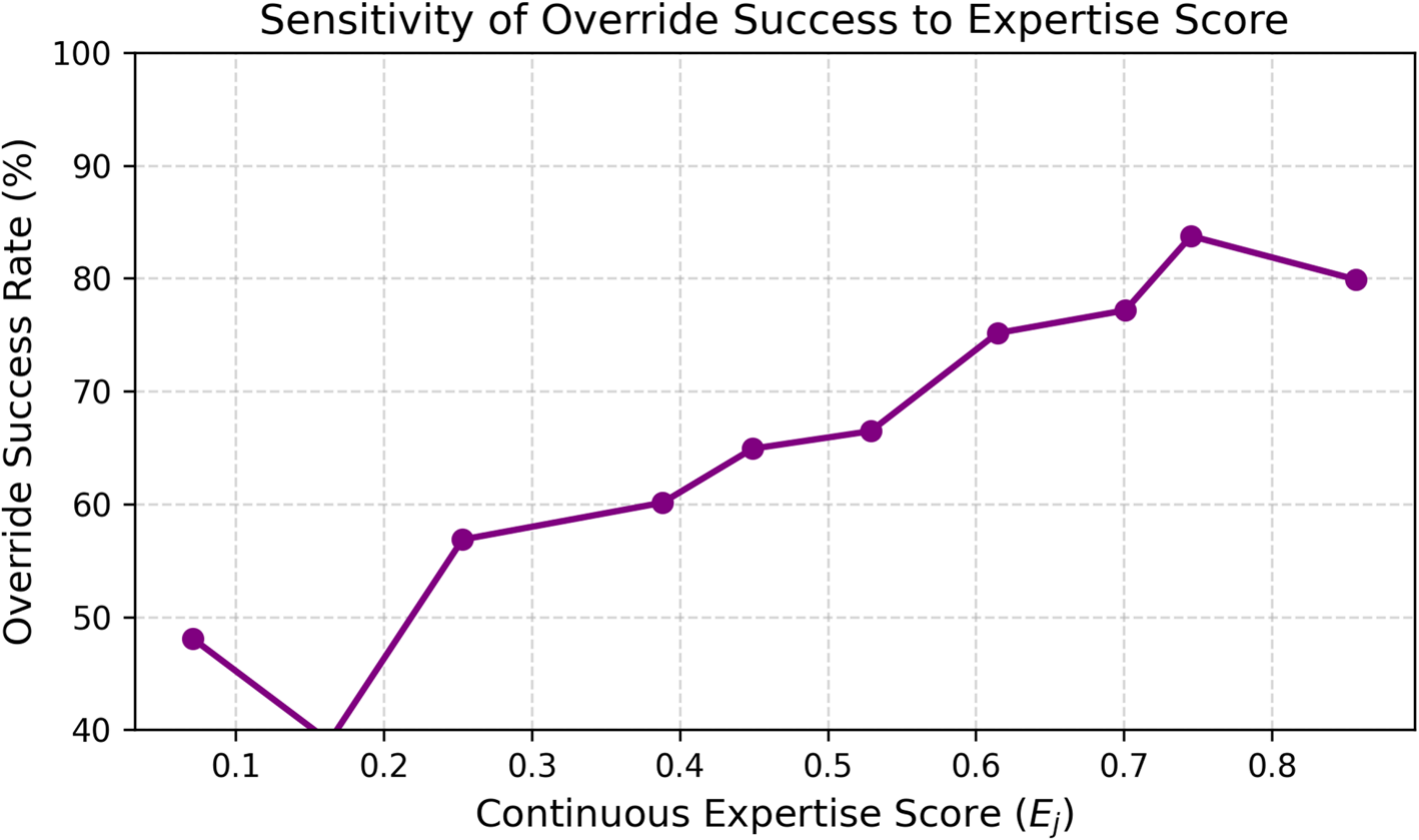
Empirical sensitivity of override success rate as a function of the continuous expertise score ($E_j$).

*Sensitivity analysis and parameter validation.* Figure 3 illustrates the sensitivity of override success rates to continuous expertise scores. As expertise $E_j$ increases, override effectiveness increases near-linearly, verifying that the continuous skill representation serves as a proxy for decision quality.

**Fig. 4:**
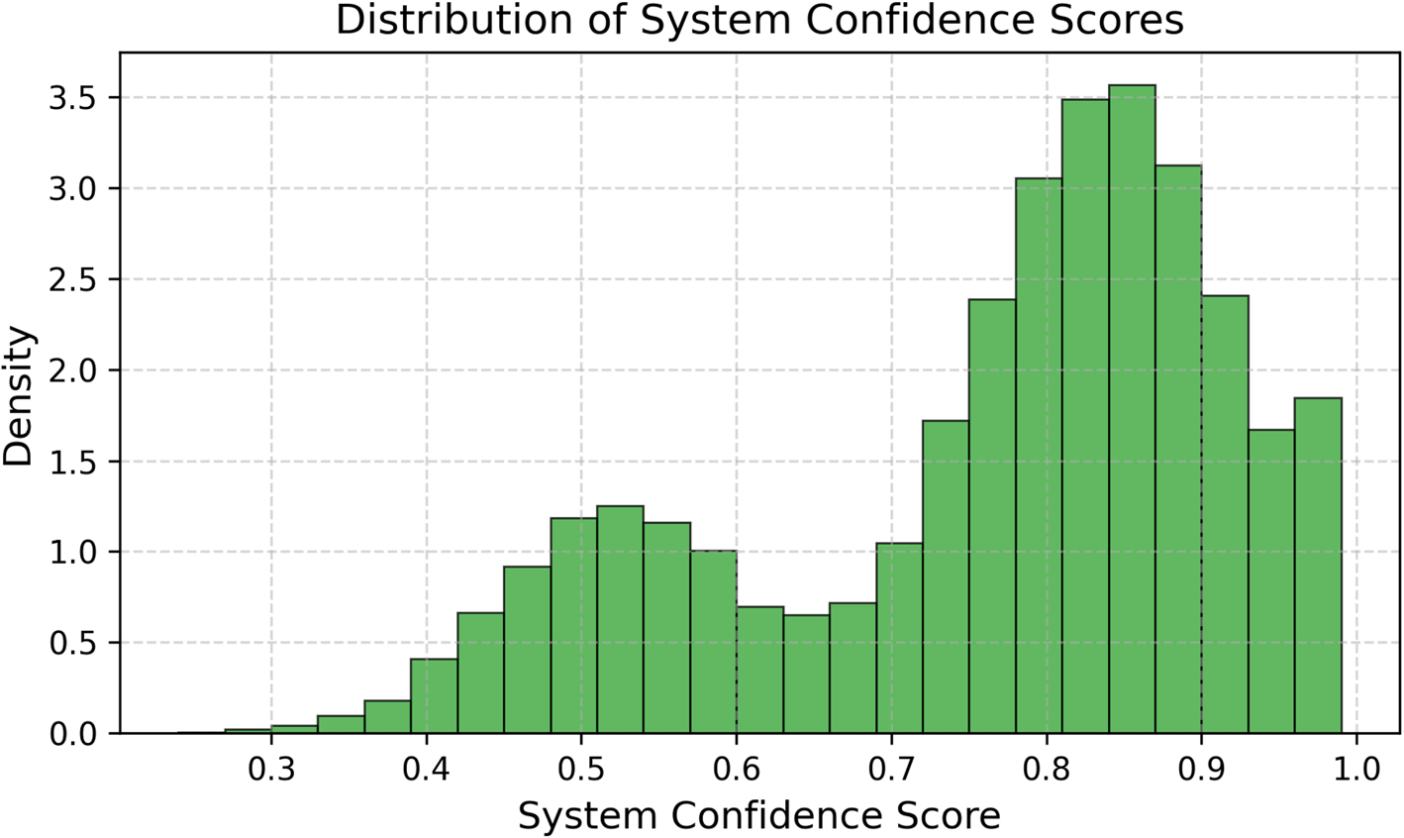
Empirical distribution of system confidence scores in HCCD-DS v2.

*Distribution of system confidence*. Confidence scores are concentrated around moderate-to-high levels without collapsing toward extreme certainty, as shown in Figure 4. This supports non-trivial human–AI interaction by keeping decision difficulty balanced.

*Machine learning benchmarks* Standard and advanced machine learning models were trained on the training split and evaluated on the test split. We evaluate Logistic Regression, Random Forest, Gradient Boosting, and a Multi-Layer Perceptron (MLP) alongside a majority baseline in Table 9.

**Table 9:** Benchmarking results for predicting human decision and final outcome (Test Split).

	Task 1: human decision			Task 2: final outcome		
Model	Accuracy	F1 (Weighted)	F1 (Macro)	Accuracy	F1 (Weighted)	F1 (Macro)
Majority baseline	0.9023	0.8560	0.4743	0.7480	0.6402	0.4279
Logistic regression	0.6927	0.7538	0.5478	0.7050	0.7214	0.6604
Random forest	0.9017	0.8557	0.4741	0.7797	0.7482	0.6334
Gradient boosting	0.9020	0.8577	0.4842	0.7807	0.7568	0.6516
MLP classifier	0.8577	0.8473	0.5384	0.7373	0.7312	0.6358

*Feature ablation study*. To establish feature predictiveness, we run a systematic ablation study using the Random Forest classifier in Table 10. The F1-score for predicting final outcomes increases monotonically as confidence, expertise, sequential trust, and XAI variables are introduced.

**Table 10:** Feature ablation analysis for predicting human decisions and final outcomes.

Feature set	Decision F1 (W)	Decision F1 (M)	Outcome F1 (W)	Outcome F1 (M)
Context Only	0.8563	0.4885	0.6791	0.5333
Context + Confidence	0.8555	0.4741	0.6982	0.5542
Context + Conf + Expertise	0.8566	0.4806	0.7020	0.5559
Context + Conf + Exp + Trust	0.8563	0.4775	0.7403	0.6227
Full Model (+ XAI)	0.8557	0.4741	0.7482	0.6334

*Parameter robustness sweep* We sweep key trust adaptation parameters ($\eta _+, \eta _-$) to assess framework robustness, summarized in Table 11. Overall success rates and F1 scores remain stable, indicating that the framework behavior is robust across parameters.

**Table 11:** Robustness sweep of trust adaptation parameters (10,000 instances).

Parameter setting	Override rate (%)	Success rate	Decision F1 (Weighted)
Base settings ($\eta _+=0.05, \eta _-=0.15$)	9.09	75.69	0.8685
Low trust decay ($\eta _-=0.08$)	8.57	75.51	0.8795
High trust decay ($\eta _-=0.25$)	10.39	76.31	0.8599
Symmetric trust ($\eta _+=0.10, \eta _-=0.10$)	7.18	76.05	0.9050

**Fig. 5:**
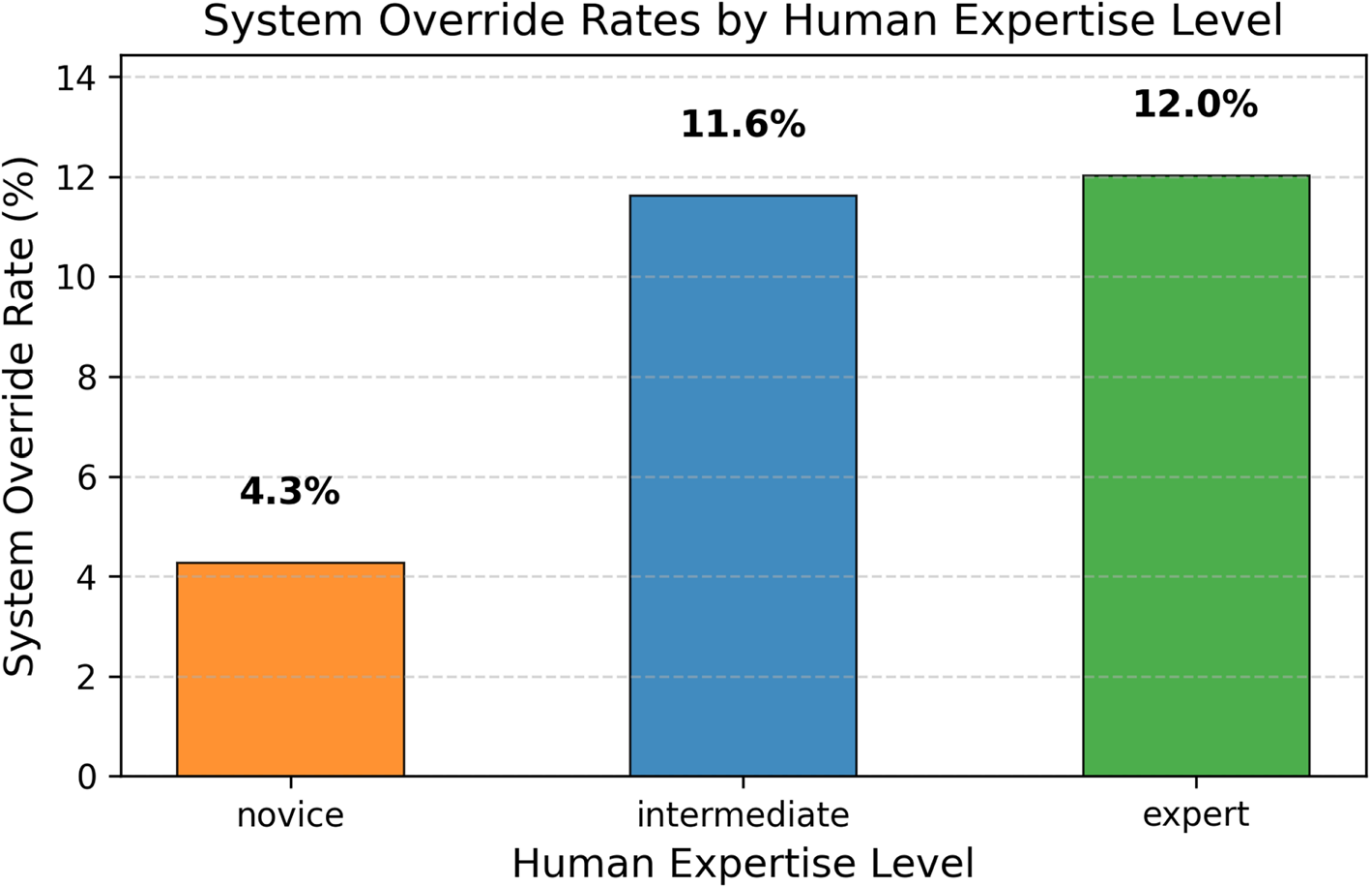
System override rates by human expertise level in HCCD-DS v2.

**Fig. 6:**
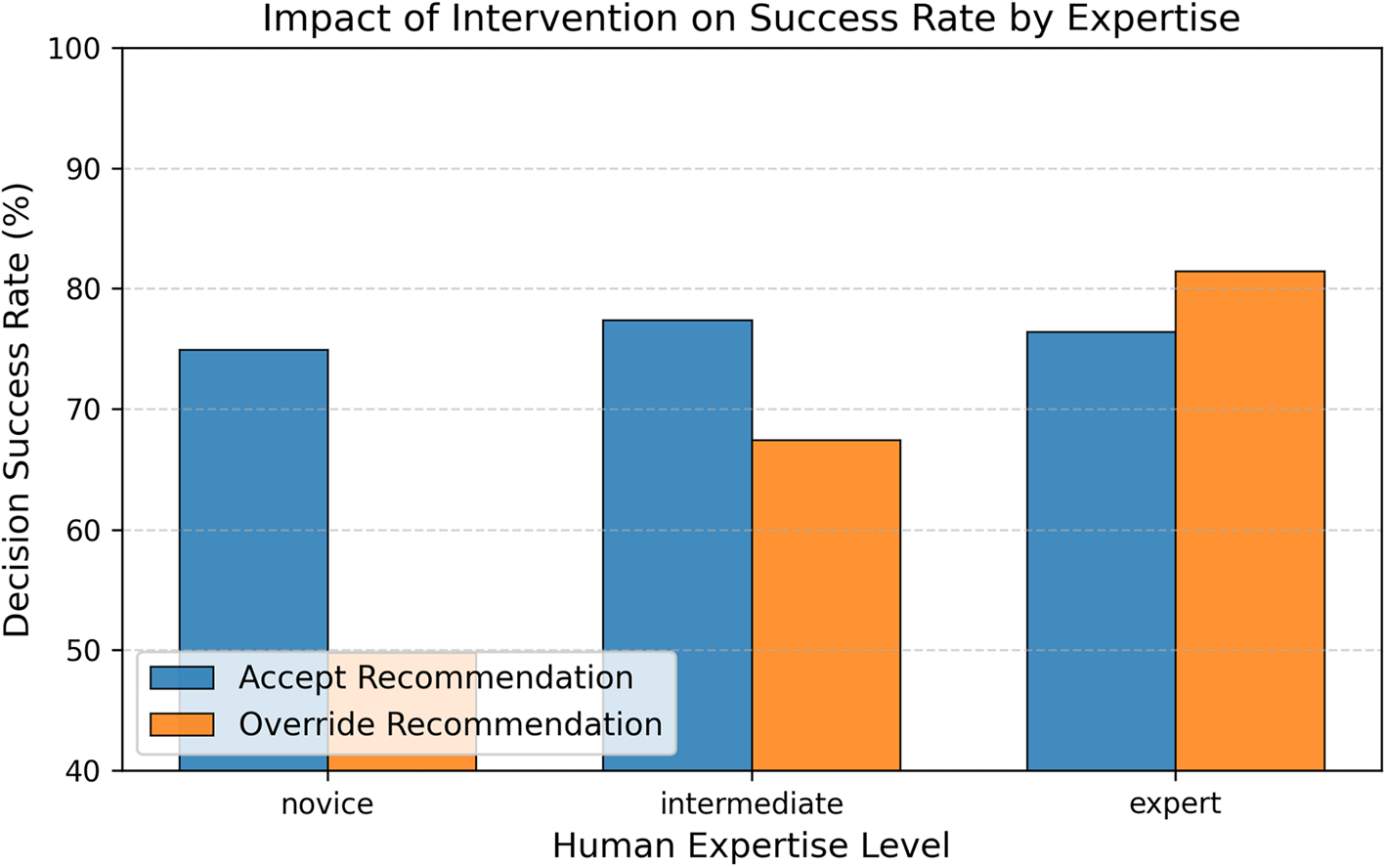
Impact of human intervention on decision success for novice and expert users.

*Expertise-driven behavioral validation*. Figure 5 and Figure 6 further illustrate the override rates and success impacts. As shown, expert overrides are beneficial, while novice overrides decrease success, confirming the intended expertise dynamics.

### Recoverability of simulated behavioral policies and circular validation risk

A key criticism of simulated human–AI datasets is the risk of circular validation. If human decisions are generated using simple, deterministic functions of the context and confidence scores, machine learning models can trivially reconstruct (or recover) the generator’s underlying behavioral policy. In the original HCCD-DS release, predicting human decisions (Task 1) achieved an F1-score of 0.95, which reflected the recovery of simulator rules rather than realistic predictive modeling.

In HCCD-DS v2, we mitigate this circularity by introducing continuous expertise, individual risk tolerance biases, and sequential trust adaptation. Because trust behaves as a latent state variable that changes dynamically over time based on the history of previous system successes and failures, the human decision at step *t* is no longer conditionally independent of previous steps. Standard static classifiers (which only inspect the current instance features) struggle to predict the minority override class. As shown in Table 9, the weighted F1 remains high (around 0.85) and close to the majority baseline of 0.8560 because accept decisions dominate the class distribution (approximately 91% of instances). Therefore, macro F1 is much more informative for evaluating the minority override behavior. The standard models achieve macro F1 scores of only 0.47–0.54, demonstrating that the underlying behavioral policy is no longer trivially recoverable from a small set of static variables. This class imbalance and the dynamic sequential dependence reduce circular validation risk, providing a more challenging and realistic benchmark for interactive machine learning.

### Ethical considerations and responsible use of synthetic human behavior

The use of synthetic human decision logs raises ethical and practical considerations. HCCD-DS v2 is designed under explicit behavioral assumptions (algorithm aversion, trust calibration, and continuous expertise value) that align with empirical human factors research. However, we do not claim that HCCD-DS v2 replaces real-world human-AI interaction logs.

Instead, HCCD-DS v2 should be positioned as a transparent, configurable benchmark for controlled testing of interaction-aware DSS methods prior to deploying models with real human practitioners. When using this dataset to evaluate deferral algorithms or trust calibration policies, researchers must acknowledge that simulated users reflect stylized, averaged populations and cannot capture the full spectrum of cognitive factors (e.g., fatigue, organizational pressure, learning shifts, and individual biases) present in actual clinical, cybersecurity, or industrial settings.

## Discussion and limitations

This section discusses the implications of the findings derived from HCCD-DS v2 and outlines the limitations that should be considered when interpreting the dataset and its potential applications. Rather than emphasizing predictive performance, the discussion focuses on what the observed behavioral patterns reveal about human–AI collaboration in decision support systems and how the dataset can be used to advance future research.

The analysis results indicate that HCCD-DS v2 captures decision-making behavior in a manner that goes beyond model-centric benchmarks. In particular, the dataset highlights that human intervention is not uniformly beneficial. Expert users intervene more frequently and, when they do, their interventions tend to improve decision outcomes. In contrast, novice interventions are comparatively rare and are more likely to degrade performance when they occur. This conditional contribution of human expertise reinforces the importance of explicitly modeling user skill and experience, rather than assuming that human oversight automatically improves system performance.

These findings have direct implications for the design and evaluation of human-in-the-loop decision support systems. DSS architectures that rely on unconditional human validation may overestimate the value of intervention and underestimate the risks associated with miscalibrated trust or insufficient expertise. By contrast, the behavioral patterns encoded in HCCD-DS v2 support the study of adaptive interaction strategies, such as selectively requesting human input based on contextual risk, system confidence, or expertise level.

Another notable observation concerns the relationship between system confidence and contextual risk. The weak association between these variables reflects plausible operational conditions in which model confidence is often calibrated with respect to internal uncertainty but poorly aligned with external or environmental risk factors. In complex and uncertain operational settings, maintaining robust machine learning performance requires models that can dynamically adapt to noise, uncertainty, spatial irregularity, and domain-specific operating conditions, a challenge also emphasized in recent robust AI studies such as DSYOLO^25^. By explicitly encoding confidence, risk, and explainability metadata at the decision-instance level, HCCD-DS v2 enables systematic investigation of trust calibration, explanation-aware interaction policies, and the timing of human intervention.

From an explainable AI perspective, the dataset supports research that moves beyond post-hoc interpretation of model outputs. Because explanations are directly linked to individual decision instances and associated human responses, HCCD-DS v2 can be used to examine how explanations influence acceptance, override behavior, and eventual outcomes. This structure allows researchers to explore explanation usefulness and human reliance in a controlled yet behaviorally plausible setting.

The benchmarking results further clarify the analytical role of the dataset. Human decision behavior is highly predictable from contextual and expertise-related features, whereas final decision outcomes are deliberately difficult to predict. This asymmetry is a defining characteristic rather than a limitation. It reflects the inherent uncertainty of real-world decision-making, where outcomes depend on stochastic factors and nuanced human judgment that cannot be trivially optimized by a single predictive model. As a result, HCCD-DS v2 is well suited for evaluating decision support approaches that emphasize collaboration, uncertainty management, and explainability rather than end-to-end automation.

Despite these strengths, several limitations should be acknowledged. First, HCCD-DS v2 is generated through a simulation framework rather than collected from real-world operational logs. Although the generation logic is grounded in established human–AI interaction literature and validated through sensitivity analysis and benchmarking, simulated behavior may not capture all subtleties of real human decision-making. Consequently, findings derived from the dataset should be interpreted as indicative patterns rather than definitive empirical observations.

Second, while user expertise is modeled as a continuous parameter, our simulation assumes it remains static for a given user throughout the session. In practice, human expertise is dynamic, context-dependent, and influenced by learning shifts, cognitive fatigue, and organizational pressure over time. While the current representation enables structured evaluation and reproducible benchmarks, it simplifies the complex and evolving spectrum of human skill.

Finally, the application domains represented in HCCD-DS v2 are intentionally generic to support cross-domain applicability. Domain-specific regulations, workflows, and operational constraints are not explicitly modeled. Researchers interested in highly specialized decision environments may therefore need to adapt or extend the dataset generation framework to better reflect domain-specific realities.

Overall, HCCD-DS v2 provides a structured benchmark for studying interaction-aware decision support under uncertainty. By jointly modeling recommendation confidence, contextual risk, user expertise, and intervention outcomes, the dataset supports evaluation of computational approaches that go beyond prediction-only settings toward plausible human–AI decision workflows.

## Conclusion

This paper introduced HCCD-DS v2, a transparent synthetic benchmark dataset for human–AI decision support under contextual uncertainty. Compared with the original release, the revised benchmark incorporates continuous expertise, sequential trust adaptation, individual risk tolerance, explanation-sensitive reliance, and domain-specific scenario profiles.

The empirical results show that intervention utility is conditional rather than uniform: expert overrides are more often beneficial, while novice overrides are more frequently harmful. Benchmark analyses further indicate that interaction behavior is easier to model than final outcomes, consistent with the stochastic nature of high-stakes decision environments.

These properties make HCCD-DS v2 useful for evaluating computational methods that optimize collaboration quality, trust calibration, and intervention timing, instead of optimizing predictive accuracy alone. The dataset therefore provides a practical foundation for reproducible research on robust and explainable decision support pipelines.

## Data Availability

The HCCD-DS v2 dataset, including all synthetic decision instances across healthcare, cybersecurity, and IoT domains, is publicly available via Zenodo at https://doi.org/10.5281/zenodo.20557057. All data generated or analysed during this study are included in this published article (and its supplementary information files).

## Code availability

The computational pipeline used for synthetic data generation, preprocessing, and benchmark evaluation is publicly available on Zenodo (https://doi.org/10.5281/zenodo.20557057) and maintained as an open-source repository under the MIT License. Scripts to reproduce all tables, figures, and benchmark metrics reported in this study are provided.
